# Impact of chemotherapy on sexual dysfunction in Turkish women with breast cancer: A single-center prospective cohort study

**DOI:** 10.1097/MD.0000000000043516

**Published:** 2025-07-18

**Authors:** Ruhper Çekin, Didar Şenocak, Serdar Arici

**Affiliations:** a Department of Oncology, Okan University Hospital, Istanbul, Türkiye; b Department of Oncology, University of Health Sciences, Sultan Abdulhamid Khan Training and Research Hospital, Istanbul, Türkiye.

**Keywords:** adjuvant therapy, breast cancer, chemotherapy-related toxicity, sexual dysfunction

## Abstract

Although numerous studies have explored the connection between breast cancer surgery and sexual function, research on chemotherapy’s temporal effects is limited. Addressing the impact of chemotherapy on sexual dysfunction is critical for improving quality of life. The aim of this study is to investigate changes in sexual function before, during, and after chemotherapy treatment, with a focus on associated factors. A total of 101 sexually active, reproductive-aged women diagnosed with locally advanced breast cancer were included in the study. The sexual dysfunction was evaluated by using the female sexual function index (FSFI) across 3 treatment phases: before, during, and after chemotherapy. Covariates such as age, baseline sexual dysfunction, tumor localization, comorbidity, family history of cancer, and receptor-related factors were analyzed for their influence on score changes during specific periods. A mixed-effects model was employed to evaluate associations and interactions between these variables and sexual function outcomes. Sexual function scores significantly declined across treatment phases. Notable reductions were observed in desire (*P* < .001), arousal (*P* < .001), lubrication (*P* < .001), orgasm (*P* < .01), and satisfaction (*P* < .01), while pain scores increased (*P* < .01). Total FSFI scores significantly dropped during and after chemotherapy (*P* < .001 and *P* < .01, respectively). Patients with preexisting sexual dysfunction experienced significantly greater declines in desire, lubrication, and satisfaction, along with more pronounced increases in pain-related discomfort scores, particularly in the FSFI pain subscale (*P* < .01). Older age, comorbidity, and tumor localization were significantly associated with worsening sexual function, whereas receptor status and histopathology showed no meaningful effect. Our findings confirm a high prevalence of sexual dysfunction among women with breast cancer. These results highlight the multifaceted impact of chemotherapy on sexual function and reveal a clear temporal pattern of changes across treatment phases.

## 1. Introduction

According to the 2022 data from the World Health Organisation’s Global Cancer Observatory, breast cancer remains the most common cancer among women in Türkiye, representing 23.5% of all new cancer cases in females, with 25,249 new cases reported that year.^[1]^ The age-standardized incidence rate for breast cancer is 46.8 per 100,000 Turkish women.^[1]^ It is also the leading cause of cancer-related deaths among Turkish women, accounting for 5.7% of all cancer deaths, with 7360 fatalities in 2022 and an age-standardized mortality rate of 10.5 per 100,000 women.^[1]^ Furthermore, the 5-year prevalence of breast cancer in Türkiye stands at 100,765 cases, corresponding to a prevalence rate of 232.6 per 100,000 women.^[1]^ These statistics highlight the significant impact of breast cancer on women’s health in Türkiye and the critical need for ongoing efforts in prevention, early detection, and treatment of physiological and psychological elements.

Although female breast cancer survivors generally have a more favorable prognosis compared to other cancers, they frequently report a range of side effects, such as nausea, hair thinning or loss, hot flashes, vaginal dryness, and irregularities in menstruation.^[2–4]^ The most prevalent side effect is sexual dysfunction, which encompasses various conditions such as anorgasmia, dyspareunia, difficulties with arousal or lubrication, vaginismus, and reduced or absent libido.^[5,6]^ A 2021 study conducted in Malaysia revealed that between 31.6% and 91.2% of female breast cancer patients are likely to experience sexual dysfunction following treatment.^[7]^

The type of treatment used for breast cancer plays a crucial role in the development of sexual dysfunction, including painful intercourse, vaginal dryness, reduced sexual desire or satisfaction, and breast numbness among survivors.^[7–9]^ Endocrine therapy with aromatase inhibitors reduces estrogen levels in the blood, leading to decreased libido and symptoms like vaginal dryness and itching. Additionally, more than 50% of women receiving aromatase inhibitor therapy report experiencing dyspareunia.^[4]^ Chemotherapy, while beneficial, also has its drawbacks, including side effects such as hair loss, weight gain, gastrointestinal issues, fatigue, menstrual irregularities, and even premature ovarian failure or menopause.^[3,4,10]^ Since a woman’s sense of sexuality often relates to her physical appearance, changes such as hair loss and weight gain can diminish self-confidence, making her feel less attractive and less interested in sexual activity.^[11]^ Evidence shows that women who undergo breast-conserving surgeries, such as lumpectomies, partial mastectomies, or segmental mastectomies, have a significantly lower risk of experiencing sexual dysfunction compared to those who have a complete mastectomy.^[12]^ Given that the treatment modality for breast cancer plays a significant role in the development of sexual dysfunction among survivors, understanding and anticipating potential sexual health issues related to the chosen treatment can clearly facilitate overcoming the associated challenges.

Recent advancements in breast cancer outcomes can primarily be attributed to 2 key factors: early detection of the disease at a subclinical stage and advancements in personalized treatment approaches.^[13]^ There is an urgent need to develop strategies and resources to manage breast cancer survivors effectively. A systematic approach is essential to address long-term treatment effects, including chemotherapy-related issues, fertility preservation in premenopausal women, and overall quality of life. Additionally, implementing lifestyle interventions for survivors should be prioritized to support their recovery and well-being. Breast cancer survivorship care plans generally include nutritional counseling, physical activity programs, mental health support, fertility preservation counseling, bone health monitoring, and sexual health counseling to promote a healthier lifestyle. Because chemotherapy can be a highly stressful experience, often leading to elevated levels of anxiety and depression in cancer patients, treatment-related side effects, combined with the psychological burden of ongoing follow-up care, can significantly reduce the quality of life for these women with cancer.^[14]^ The majority of female breast cancer survivors not only aim to extend their survival but also seek to enhance their quality of life. Sexual dysfunction, a common consequence of breast cancer treatment, significantly contributes to the decline in quality of life, affecting up to 3/4 of female breast cancer survivors.^[15,16]^ As a result, addressing the rehabilitation of breast cancer patients with sexual dysfunction remains a critical concern.

In the context of Turkish traditional and cultural values, sex and sexuality remain taboo topics, although they are discussed slightly more openly today compared to previous years. Even health professionals tend to avoid addressing sexual issues. Consequently, a review of published literature on this overlooked subject revealed that only a limited number of studies on sexual dysfunction among breast cancer patients have been reported in Türkiye. Sertoz et al examined the influence of different surgical approaches on body image, sexuality, self-esteem, and marital adjustment in breast cancer patients.^[8]^ Their findings revealed that total mastectomy adversely affected self-esteem, with avoidance of sexual activity being the most commonly reported sexual dysfunction among Turkish breast cancer survivors. A study conducted by Öztürk and Akyolcu evaluated sexual function and dysfunction in Turkish women undergoing surgical treatment for breast cancer, revealing that mastectomy negatively impacted sexual function.^[9]^ These studies evaluated the effects of breast cancer surgery on sexual functions.

Longer survival rates and an increasing number of cured patients necessitate dedicated strategies to address the long-term consequences of breast cancer treatments, with a particular focus on sexual quality of life. However, the short-term and long-term effects of chemotherapy on sexual function remain insufficiently studied. In our study, we aimed to evaluate the role of chemotherapy in the pathophysiology of sexual dysfunction among women with breast cancer. Specifically, we assessed patients’ sexual function before treatment, compared the various domains of sexual dysfunction during and after treatment, and examined the relationship between sexual dysfunction and clinical as well as demographic characteristics using standardized, cross-culturally validated methods.

## 2. Materials and methods

This prospective, single-center, longitudinal cohort study was approved by the Ethics Committee of the Cemil Tascioglu City Hospital, Istanbul, Türkiye (Study Protocol Number: 2019-48670771-514.10) and was performed in accordance with the Declaration of Helsinki. Written informed consent was obtained from all participants. Women with locally advanced breast cancer who received neoadjuvant chemotherapy (NAC) at the medical oncology clinic were enrolled in the study. The demographic features and chronic diseases of reproductive-aged women with an active sexual life were recorded. The primary goal was to evaluate the changes in patients’ sexual health across the phases of chemotherapy-before, during, and after treatment-focusing on the influence of the treatment timeline and impact on their sexual well-being.

### 2.1. Participants

In this study, we aimed to analyze sexual dysfunction patterns across chemotherapy stages in women with breast cancer who visited the oncology clinic for routine follow-up between January 2020 and January 2023. A total of 148 patients who met the inclusion criteria were enrolled. However, 33 patients with breast cancer did not meet the criteria, and they were excluded from the study. In addition, 14 patients discontinued their follow-up at our clinic, making their data unavailable. As a result, the medical records of 101 participants were reviewed and included in the current study. All participants were diagnosed with breast cancer based on the International Classification of Diseases (ICD-10) criteria and confirmed through pathological examination. Data on participants’ clinical and socio-demographical characteristics, along with their sexual activities, were recorded in a database that was prepared by the researchers. The participants were questioned regarding their personal details, which were recorded in the case forms. The demographic and clinical variables assessed included age, comorbidity, family history of cancer, tumor localization, histopathology type, tumor grade, and hormonal receptor statuses (estrogen receptor (ER), progesterone receptor (PR), CERBB2, and HR_HER). The participants individually completed all inventories at the same point in their treatment timeline. Confidentiality was ensured throughout the survey, with no third parties permitted to be present in the room.

#### 2.1.1. Inclusion criteria

- Women aged 18 to 45 years with regular menstrual cycles and an active sexual life who were diagnosed with locally advanced breast cancer.
- Patients with clear medical records.
- Patients who agreed to participate in the study and complete the face-to-face survey.
- Patients who presented to the clinic for routine follow-up **within the overall study period (January 2020–January 2023**).

#### 2.1.2. Exclusion criteria

- Patients who were not of reproductive age or did not have an active sexual life.
- Patients with early-stage breast cancer who underwent surgery and received adjuvant chemotherapy or hormone therapy.
- Patients with sexual dysfunction before the diagnosis of breast cancer.
- Patients with severe liver, renal, or brain dysfunction, as well as those with other malignant tumors.
- Patients who declined to participate in the survey and had incomplete information or were lost to follow-up.

### 2.2. Study measures

#### 2.2.1. The female sexual function index

Female sexual function index (FSFI) is a brief, self-report measure of female sexual function, as described by Rosen et al.^[17]^ It is suggested as the “gold standard” to assess the female sexual function.^[18]^ The FSFI questionnaire is used for assessing sexual function across 6 subdomains: sexual desire, arousal, lubrication, orgasm, satisfaction, and pain. The score is determined using a 6-point Likert scale, with the total score calculated by summing the values from each domain and applying a correction factor. A cutoff score of ≤ 26 was classified as the presence of sexual dysfunction. A validation study of this questionnaire has been done in Türkiye by Aygin and Aslan.^[19]^ Scores for each domain, as well as the total FSFI scores, were calculated at 3 time points: prior to NAC (baseline), following anthracycline-based chemotherapy (AC), and 6 months after completing NAC.

### 2.3. Data collection and study design

Sociodemographic characteristics, marital status, and clinical history were gathered through patient interviews and a review of their medical records. Sexual function was evaluated at 3 different time points using the FSFI questionnaire. The assessments were conducted before the initiation of NAC as a baseline measure, after completing 4 cycles of AC but prior to commencing taxane-based regimens, and finally, 6 months following the completion of the entire NAC treatment. Additionally, participants in the study were categorized based on the presence or absence of sexual dysfunction, as determined by their initial scores on the FSFI questionnaire, and changes in their sexual function during and after the treatment process were evaluated. Surveys were administered face-to-face by the same clinician to ensure consistency and accuracy.

### 2.4. Statistical analysis

All statistical analyses were conducted using R (version 4.4.0, released in 2020), an open-source software environment for statistical computing and graphics developed by the R Foundation for Statistical Computing. Mixed-effects modeling was performed with the lme4 package. Descriptive statistics were used to summarize demographic, clinical, and outcome data. Continuous variables were reported as means and standard deviations, while categorical variables were summarized as counts and percentages. To examine the effects of treatment and covariates on sexual function scores, a linear mixed-effects model was employed. This approach was chosen due to its ability to handle correlated measurements within individuals over time and account for between-subject variability. Interactions between the treatment period and significant covariates were tested to assess the influence of clinical and demographic factors on changes in scores over time. Pairwise comparisons between treatment periods were conducted using Bonferroni-adjusted paired t-tests to identify specific time points with significant differences. These post hoc analyses were used to provide detailed insights into how sexual function scores evolved over the 3 treatment periods. A *P*-value of < .05 was considered statistically significant.

## 3. Results

The study included 101 participants with a mean age of 39.1 years (SD = 4.73; range: 26–45 years). Comorbidities were present in 9.9% of the patients (n = 10), while 90.1% (n = 91) had no comorbid conditions. A positive family history of breast cancer was reported in 35.6% (n = 36) of the participants, whereas 64.4% (n = 65) reported no family history. Tumors were right-sided in 47.5% (n = 48) of patients and left-sided in 52.5% (n = 53). Histopathological analysis revealed ductal in 87.1% (n = 88) of cases, with the remaining 12.9% (n = 13) classified as lobular or others. The tumors were graded as follows: Grade 1 in 30.7% (n = 31), Grade 2 in 49.5% (n = 50), and Grade 3 in 19.8% (n = 20). Regarding hormone receptor status, ER positivity was observed in 59.4% (n = 60) of patients, and PR positivity was detected in 44.6% (n = 45). HER-2 (CERBB2) positivity was present in 41.6% (n = 42) of cases. Hormone receptor and HER-2 (HR-HER) positivity was identified in 33.7% (n = 34) of the patients, while 66.3% (n = 67) were negative for this biomarker. Table 1 illustrates the demographic and clinical characteristics of patients.

**Table 1:** The demographic and clinical characteristics of patients with breast cancer.

Characteristics	Values
Age (yr)	39.1 ± 4.73 (min: 26, max: 45)
Comorbidity	Present: 10 (9.9%), absent: 91 (90.1%)
Family history of cancer	Present: 36 (35.6%), absent: 65 (64.4%)
Localization of tumor	Right: 48 (47.5%), left: 53 (52.5%)
Histopathology	Ductal: 88 (87.1%), others: 13 (12.9%)
Tumor grade	Grade 1: 31 (30.7%), grade 2: 50 (49.5%), grade 3: 20 (19.8%)
Estrogen receptor	Positive: 60 (59.4%), negative: 41 (40.6%)
Progesterone receptor	Positive: 45 (44.6%), negative: 56 (55.4%)
CERBB2	Positive: 42 (41.6%), negative: 59 (58.4%)
HR_HER	Positive: 34 (33.7%), negative: 67 (66.3%)

The mixed-effects model revealed significant changes in sexual function scores across the 3 treatment periods (before, during, and after chemotherapy). Statistically significant differences were observed across all subscales. Sexual desire scores declined from 3.97 ± 1.07 at baseline to 2.08 ± 1.13 after treatment, with a mean difference of −1.89 (95% CI: −2.10 to −1.68). Arousal declined from 4.28 ± 1.24 to 1.74 ± 1.60, with a mean difference of −2.54 (95% CI: −2.82 to −2.26). Lubrication scores dropped from 5.18 ± 1.23 to 2.10 ± 1.91, reflecting a mean difference of −3.08 (95% CI: −3.44 to −2.72). Orgasm scores fell from 4.17 ± 1.14 to 1.93 ± 1.82, with a mean difference of −2.24 (95% CI: −2.59 to −1.89). Satisfaction declined from 4.34 ± 1.12 to 1.91 ± 1.84, with a mean difference of −2.43 (95% CI: −2.75 to −2.11). Pain/discomfort scores decreased from 4.93 ± 1.31 to 2.27 ± 2.18, with a mean difference of −2.66 (95% CI: −3.04 to −2.28). The total FSFI score dropped significantly from 26.9 ± 5.26 at baseline to 12.0 ± 9.70 after treatment, corresponding to a mean difference of −14.9 (95% CI: −16.7 to −13.1). All pairwise comparisons were statistically significant (*P* < .001). Table 2 provides the changes in sexual function subscores and total scores over time.

**Table 2:** The changes in FSFI questionnaire scores over time.

Subscale scores (mean ± SD)	Before the treatment	During the treatment	After the treatment	*P*-value (time effect)
Sexual desire	3.97 ± 1.07	2.62 ± 1.22	2.08 ± 1.13	<.001
Arousal	4.28 ± 1.24	2.69 ± 1.67	1.74 ± 1.60	<.001
Lubrication	5.18 ± 1.23	3.39 ± 1.96	2.10 ± 1.91	<.001
Orgasm	4.17 ± 1.14	2.84 ± 1.64	1.93 ± 1.82	<.001
Satisfaction	4.34 ± 1.12	2.91 ± 1.75	1.91 ± 1.84	<.001
Pain/discomfort	4.93 ± 1.31	3.55 ± 2.07	2.97 ± 2.18	<.001
Total scale scores	26.9 ± 5.26	18.0 ± 9.32	12.0 ± 9.70	<.001

FSFI = female sexual function index, SD = standard deviation.

Subgroup analysis based on baseline sexual dysfunction status revealed further differences. After treatment, patients with preexisting sexual dysfunction had lower FSFI scores than those without (*P* < .01). The between-group mean difference in total score was + 4.0 (95% CI: +2.6 to + 5.4). Similar trends were seen in desire (+0.75; 95% CI: +0.53 to + 0.97) (*P* < .05), lubrication (+0.50; 95% CI: +0.28 to + 0.72) (*P* < .05), satisfaction (+0.35; 95% CI: +0.12 to + 0.58) (*P* < .05), and pain (-0.30; 95% CI: −0.52 to −0.08) (*P* < .01) subscales, all favoring patients without baseline dysfunction. Table 3 presents the FSFI scores of patients with and without preexisting sexual dysfunction across treatment phases.

**Table 3:** The interaction effects from the linear mixed-effects model, testing the influence of preexisting sexual dysfunction on changes in sexual function scores across treatment periods.

Subscale scores (mean ± standard deviation)	Patients with preexisting SD, before the treatment	Patients with preexisting SD, during the treatment	Patients with preexisting SD, after the treatment	Patients without preexisting SD, before the treatment	Patients without preexisting SD, During The Treatment	Patients without preexisting SD, After The Treatment	*P*-value (interaction)
Sexual desire	3.30 ± 0.90	2.10 ± 0.98	1.50 ± 0.83	4.25 ± 0.95	2.95 ± 1.18	2.25 ± 1.07	.002
Arousal	3.85 ± 0.98	2.35 ± 1.27	1.35 ± 0.73	4.55 ± 1.10	3.05 ± 1.32	2.00 ± 1.22	.001
Lubrication	4.50 ± 1.01	2.95 ± 1.30	1.80 ± 1.10	5.60 ± 0.85	3.85 ± 1.40	2.30 ± 1.50	.001
Orgasm	4.00 ± 1.20	2.70 ± 1.45	1.80 ± 1.05	4.20 ± 0.88	3.20 ± 1.15	2.05 ± 1.15	.010
Satisfaction	4.05 ± 1.10	2.65 ± 1.35	1.75 ± 0.95	4.55 ± 1.05	3.05 ± 1.32	2.10 ± 1.18	.008
Pain/discomfort	5.00 ± 1.20	3.80 ± 1.50	2.50 ± 1.40	4.90 ± 1.05	3.30 ± 1.20	2.20 ± 1.15	.015
Total scale scores	20.0 ± 3.50	15.0 ± 5.10	10.0 ± 4.80	29.0 ± 4.50	21.0 ± 6.00	14.0 ± 6.50	<.001

SD = standard deviation.

The mixed-effects model was employed to examine the influence of demographic and clinical covariates on changes in scores across treatment periods (before, during, and after the treatment). The analysis revealed several significant associations and interaction effects related to demographic and clinical variables. Older age was significantly associated with lower scores across all periods (*P* = .04), indicating an independent impact of age on outcomes. However, interaction effects between periods were not significant, suggesting that changes in scores over time did not vary by age. No significant fixed effect was observed for comorbidity status (*P* = .10). However, interaction effects were significant between the before and after the treatment periods (*P* = .01) and showed a trend toward significance in the during and after the treatment periods (*P* = .05), indicating that comorbidities influenced changes in scores during specific phases. Family history of cancer had no significant fixed effect on scores (*P* = .45). However, a significant interaction effect was observed during and after the treatment periods (*P* = .04), suggesting that family history of cancer influenced score changes during this phase. Tumour localization had a significant fixed effect on baseline scores (*P* = .02). Interaction effects were significant between the before-and-after periods (*P* = .05), indicating that tumor localization impacted score changes during this time frame, but not during and after the treatment period (*P* = .11). No significant fixed effect was observed for histopathology (*P* = .62). Interaction effects were also nonsignificant across all periods (Before-After: *P* = .90; During-After: *P* = .87). Tumour grade had no significant fixed effect (*P* = .16). Interaction effects showed no significance for the Before-After period (*P* = .27) but a trend toward significance for the During-After period (*P* = .08). Neither ER nor PR status showed significant fixed effects (ER: *P* = .56; PR: *P* = .77) or interaction effects across periods. Similarly, HER-2 (CERBB2) status and combined hormonal receptor and HER-2 (HR_HER) status showed no significant fixed effects (HER-2: *P* = .76; HR_HER: *P* = .70) or interaction effects (HER-2 Before-After: *P* = .82; HER-2 During-After: *P* = .72; HR_HER Before-After: *P* = .83; HR_HER During-After: *P* = .55). A significant fixed effect was observed for patients with baseline sexual dysfunction (*P* = .00), indicating its independent impact on outcomes, while interaction effects across treatment periods were not significant (Before-After: *P* = .13; During-After: *P* = .33). Table 4 shows the variability in treatment responses regarding sexual function, influenced by individual covariates.

**Table 4:** The fixed effects, including main and interaction effects, from the linear mixed-effects model examining changes in sexual function scores over treatment periods.

Covariate	Fixed effect (*P*-value)	Interaction effects (period × covariate *P*-value)
Age	.04*	Before–after: .28
		During–after: .56
Comorbidity	.10	Before–after: .01*
		During–after: .05
Family history	.45	Before–after: .26
		During–after: .04*
Localization	.02*	Before–after: .05*
		During–after: .11
Histopathology	.62	Before–after: .90
		During–after: .87
Tumor grade	.16	Before–after: .27
		During–after: .08
Estrogen receptor	.56	Before–after: .74
		During–after: .19
Progesterone receptor	.77	Before–after: .77
		During–after: .82
CERBB2 (HER-2 status)	.76	Before–after: .82
		During–after: .72
HR_HER (hormonal receptor and HER-2)	.70	Before–after: .83
		During–after: .55
Patients with preexisting sexual dysfunction	.00*	Before–after: .13
		During–after: .33

* *P* < .05.

## 4. Discussion

The aim of the current study was to evaluate the impact of chemotherapy on sexual function in women with breast cancer. Using standardized and validated instruments, we assessed changes in sexual health across treatment phases, before, during, and after chemotherapy, focusing on the effects of treatment sequence and timing. The study included women aged ≥ 18 years with histopathologically confirmed breast cancer, excluding those with terminal conditions or severe comorbidities. The prevalence of sexual dysfunction symptoms in breast cancer patients, along with their patterns and associated factors, was investigated. The study revealed significant changes in sexual function scores across treatment phases (before, during, and after chemotherapy), with notable declines observed in desire, arousal, lubrication, orgasm, and satisfaction, while pain scores increased. Patients with baseline sexual dysfunction experienced steeper declines in sexual function and greater increases in pain during and after treatment compared to those without dysfunction. The clinic and demographical variables, including age, comorbidity, and tumor localization, were identified as significant factors influencing sexual function outcomes, while ER, PR, and HER-2 receptor status and histopathology showed no significant effects. These findings underscore the dynamic impact of chemotherapy on sexual health and highlight the importance of subject’s features in shaping treatment outcomes on sexual function.

Our analysis revealed that older age, comorbidities, and tumor localization were significant covariates associated with increased sexual dysfunction, particularly affecting domains such as desire, lubrication, satisfaction, and pain. For instance, older women demonstrated consistently lower FSFI scores across all phases of treatment, likely reflecting a combination of age-related physiological decline and the cumulative burden of chemotherapy-induced side effects. Comorbid conditions appeared to further exacerbate pain and reduce satisfaction, while tumor laterality influenced baseline function and score changes over time. In contrast, receptor status and histopathology did not show a significant association with sexual outcomes. While a cross-sectional study by Ooi et al^[6]^ identified family history of breast cancer as a factor influencing sexual dysfunction following surgery, our findings suggest that chemotherapy-related effects, rather than surgical or genetic factors, may play a more prominent role in shaping sexual health trajectories. Despite methodological differences, these comparisons underscore the multifactorial nature of sexual dysfunction in breast cancer and support the need for tailored clinical interventions based on individual risk profiles.

Although the present study did not include a qualitative component, cultural factors may help contextualize the high baseline prevalence and severity of sexual dysfunction observed among participants. There are notable regional and study-specific variations in the frequency of sexual dysfunction in patients with breast cancer. For instance, studies from Malaysia report a prevalence of 31.6% to 91.2%, highlighting the wide range due to methodological and cultural differences.^[6]^ A recent systematic review reported that 30% to 80% of women with gynecological or breast cancer experience sexual dysfunction.^[20]^ Additionally, these women face a 2.7 to 3.5-fold higher risk of developing sexual dysfunction, regardless of its type. A strong predictor of greater reductions in sexual function after chemotherapy was the high incidence of baseline sexual dysfunction that we identified in our study. This may be due to cultural and ethnic reasons, as Turkish society is generally regarded as holding traditional and conservative values, where open discussions of sexuality may be considered culturally sensitive. These cultural dynamics might contribute to increased psychological and social challenges related to sexual dysfunction, although this interpretation remains speculative in the absence of supporting sociocultural data.

Sexual dysfunction in cancer patients is not solely attributed to the anatomical location of the tumor but may also result from physiological changes caused by the side effects of cancer treatments.^[20]^ Our findings confirm that chemotherapy has a substantial and multifaceted impact on female sexual function, with significant declines observed across all FSFI domains, particularly in desire, arousal, lubrication, orgasm, and satisfaction. Pain-related discomfort also increased notably following treatment. These results support existing literature suggesting that chemotherapeutic regimens, especially those involving anthracyclines, contribute to premature ovarian failure, hormonal fluctuations, and psychological stress, all of which can impair sexual health. Our results are consistent with studies by Papadopoulou et al^[14]^ and Park et al^[15]^ which also found chemotherapy to be a significant contributing factor to declining sexual health. Our study’s merits, however, are in assessing sexual function during 3 different treatment stages, which enables us to more thoroughly document the temporal impacts. A comprehensive approach of how sexual dysfunction changes over the course of treatment is provided by this longitudinal method, which is something that cross-sectional studies sometimes overlook.

Endocrine therapy is predominantly used in patients with hormone receptor-positive breast cancer, either as adjuvant treatment to prevent recurrence and metastasis following surgery or as a salvage therapy in cases of recurrence and metastasis.^[21]^ By lowering estrogen levels or blocking the interaction between estrogen and hormone receptors, endocrine therapy effectively inhibits estrogen-dependent breast cancer cell proliferation and tumor growth.^[21,22]^ However, it is also associated with menopausal symptoms, such as hot flushes, sleep disturbances, and vaginal dryness or atrophy. In contrast to endocrine therapy, which has been widely studied for its estrogen-depleting effects, the temporal impact of chemotherapy on sexual function remains under-investigated. By evaluating sexual function at 3 key points, before, during, and after chemotherapy, our study provides valuable longitudinal evidence of these effects, distinct from those of hormonal agents. In our study, patients receiving endocrine therapy were excluded to ensure a consistent clinical background among participants. Our results demonstrated that receptor status did not have a significant impact on sexual function. Additionally, excluding patients receiving endocrine therapy further strengthened the internal validity of our findings and allowed us to attribute observed changes specifically to chemotherapy exposure. This observation could serve as a basis for future studies evaluating the impact of endocrine therapy on sexual health.

A key strength of our study is the inclusion of participants with similar demographic and clinical profiles, as it improves the internal validity of our conclusions. Furthermore, the use of validated instruments like the FSFI ensures robust and reliable assessment of sexual function. In addition, a causal relationship was established since we used a longitudinal cohort study design. However, our study has some limitations. For example, the absence of a control group without chemotherapy limits the ability to isolate chemotherapy-specific effects. Furthermore, the self-reported nature of the FSFI may introduce reporting biases due to cultural stigmas associated with sexuality in Türkiye. In order to overcome these constraints, future research should include control groups and qualitative insights to complement quantitative data.

## 5. Conclusion

In summary, our research emphasizes the considerable and multifaceted effects of chemotherapy on sexual function in women diagnosed with breast cancer. These results highlight the necessity for healthcare providers to routinely include sexual function evaluations, such as the FSFI, in standard oncology treatment protocols, especially for younger patients and those with additional health issues. Furthermore, the hesitance of patients to bring up sexual matters indicates a pressing requirement for culturally aware sexual health counseling and education. From a policy standpoint, the incorporation of sexual health services into survivorship care plans and training programs for clinicians could improve patient outcomes. Future studies should investigate qualitative perspectives and culturally specific interventions to meet the intricate needs of varied patient groups.

## Acknowledgments

We would like to thank the participants and the our department’s staff for their contributions to this study.

## Author contributions

**Conceptualization:** Ruhper Çekin, Serdar Arici.

**Formal analysis:** Ruhper Çekin.

**Investigation:** Ruhper Çekin.

**Methodology:** Ruhper Çekin.

**Resources:** Ruhper Çekin, Didar Şenocak.

**Supervision:** Serdar Arici.

**Validation:** Ruhper Çekin.

**Writing – original draft:** Ruhper Çekin.

**Writing – review & editing:** Ruhper Çekin, Didar Şenocak, Serdar Arici.
